# Association between Shiga Toxin–Producing *Escherichia coli* O157:H7 *stx* Gene Subtype and Disease Severity, England, 2009–2019

**DOI:** 10.3201/eid2610.200319

**Published:** 2020-10

**Authors:** Lisa Byrne, Natalie Adams, Claire Jenkins

**Affiliations:** Public Health England, London, UK

**Keywords:** Shiga-toxigenic Escherichia coli, risk factors, Escherichia coli infections, epidemiology, hemolytic-uremic syndrome, bacteria, England, STEC, Shiga toxin–producing Escherichia coli, O157:H7

## Abstract

Signs and symptoms of Shiga toxin–producing *Escherichia coli* (STEC) serogroup O157:H7 infection range from mild gastrointestinal to bloody diarrhea and hemolytic uremic syndrome (HUS). We assessed the association between Shiga toxin gene (*stx*) subtype and disease severity for »3,000 patients with STEC O157:H7 in England during 2009–2019. Odds of bloody diarrhea, HUS, or both, were significantly higher for patients infected with STEC O157:H7 possessing *stx2a* only or *stx2a* combined with other *stx* subtypes. Odds of severe signs/symptoms were significantly higher for isolates encoding *stx2a* only and belonging to sublineage Ic and lineage I/II than for those encoding *stx2a* only and belonging to sublineage IIb, indicating that *stx2a* is not the only driver causing HUS. Strains of STEC O157:H7 that had *stx1a* were also significantly more associated with severe disease than strains with *stx2c* only. This finding confounds public health risk assessment algorithms based on detection of *stx2* as a predictor of severe disease.

In England, infection with Shiga toxin–producing *Escherichia coli* (STEC) serogroup O157:H7 is relatively rare; »650 cases are reported each year (*1*). However, STEC O157:H7 is a pathogen of public health concern because of its potential to cause severe disease. In England, almost two thirds of case-patients reportedly experience bloody diarrhea and 5%–14% of infections progress to the severe condition of hemolytic uremic syndrome (HUS) (*2*–*6*).

Predictors of whether HUS will develop after STEC infection include pathogen and host factors. Most at risk for development of HUS after STEC infection are children; HUS is the leading cause of renal failure in children in developed countries, including the United Kingdom and the United States (*7*). Some studies have demonstrated that female sex is also associated with HUS (*2*,*8*–*10*).

The STEC pathotype is defined by the presence of the genes encoding Shiga toxin (Stx) type 1, type 2, or both, which are located on bacteriophage incorporated into the bacterial genome (*11*). Stx1 and Stx2 can be further divided into subtypes Stx1a-1d and Stx2a-2g. Previous studies have demonstrated an association between Stx subtype and disease severity; strains producing Stx2, particularly the Stx2a subtype, are more associated with severe disease and HUS (*12*–*16*). These findings have led to the development and implementation of differential case management and public health management of cases based on Stx profile–derived STEC pathotypes in England and elsewhere (*17*–*19*).

The STEC O157:H7 population has previously been delineated into 3 main lineages (I, I/II, and II) (*20*) and 7 sublineages (Ia, Ib, Ic, IIa, IIb, IIc, and I/II). When STEC O157:H7 emerged in England in the 1980s, the dominant lineage was I/II. Phylogenetic analyses in which hierarchical single-linkage clustering performed on pairwise single-nucleotide polymorphism (SNP) difference between strains was used revealed that almost all isolates belonging to lineage I/II fell within a 250 single-linkage SNP cluster, or clade. During the 1990s, sublineage I/II was replaced by a 250 single-linkage SNP clade within sublineage Ic (*20*,*21*). More recently, a decline in sublineage Ic and a concurrent increase in sublineage IIb have been observed (*14*,*22*). The emergence of each clade appears to coincide with the acquisition of phage encoding the *stx2a* gene, which, if causing more severe disease, increases the likelihood that those cases will be detected (*20*).

The evidence base for the differential public health management of STEC cases based on pathotype has been assimilated from relatively small studies, which prompted a review of the data in England. We therefore explored the association between Stx subtype, particularly the role of Stx2a, and disease severity in England for »3,000 cases of STEC O157:H7 reported in the 11-year period 2009–2019.

## Materials and Methods

### Data, Setting, and Source

For this study, we used an observational study design. In January 2009, Public Health England (PHE) implemented the National Enhanced Surveillance System for STEC (NESSS) in England. In brief, it captures standardized epidemiologic and microbiological data for all cases of STEC reported in England through an Enhanced Surveillance Questionnaire (ESQ). For each case, these data are reconciled with microbiological data in NESSS (*3*).

We included data on all STEC O157:H7 cases in England reported from January 1, 2009, through December 31, 2019, for which the patient submitted an ESQ and whose isolates had undergone whole-genome sequencing. For each case-patient, we extracted and coded as binary variables the following: clinical data on reported signs/symptoms (nonbloody diarrhea, bloody diarrhea, vomiting, nausea, abdominal pain, and fever); whether the patient was asymptomatic, hospitalized, or died; and whether HUS developed. We coded the responses as negative when clinical symptoms were blank on the ESQ and case-patients were not recorded as being asymptomatic. We also extracted age and sex of case-patients and categorized them as children (<16 years of age) or adults, according to a priori knowledge that children are most at risk for STEC infection and for disease progression to HUS. The outcome of interest was disease severity. Case-patients were coded as having severe disease if bloody diarrhea, HUS, or death were reported. Asymptomatic persons and case-patients with nonbloody diarrhea were considered to have mild disease. We linked data derived from whole-genome sequencing, including Stx subtype and lineage, to each case.

### Laboratory Methods

In England, all fecal specimens from patients with hospital-acquired and community-acquired cases of gastrointestinal disease submitted to local hospital laboratories are tested for *E. coli* O157:H7. All isolates are submitted to the PHE Gastrointestinal Bacteria Reference Unit for confirmation. Since July 2015, all isolates have been sequenced for routine surveillance (National Center for Biotechnology Information Short Read Archive Bioproject no. PRJNA248042). Therefore, we included in this study all isolates received since July 2015 from case-patients with completed ESQs. In addition, we included isolates of STEC O157:H7 submitted to the Gastrointestinal Bacteria Reference Unit from January 2009 through June 2015 and sequenced as part of previous studies from case-patients with ESQs (*20*). The process for whole-genome sequencing has been described in detail (*14*).

### Statistical Analyses

We used Stata 13.1 (StataCorp, https://www.stata.com) for our analyses. We described cases with respect to clinically mild and severe disease by patient age, sex, and Stx subtype. We used logistic regression to investigate the relationship between Stx subtype and disease severity, adjusting for age and sex. For each variable, we calculated odds ratios (ORs) for case-patients reporting severe disease compared with those reporting mild disease. We chose the Stx2c subtype as the baseline for Stx subtype because this subtype is associated with less severe disease. To further explore the phylogenetic relationships within Stx2a isolates, we used logistic regression to investigate the relationship between Stx2a sublineages and disease severity, adjusting for age and sex. For each variable, we calculated ORs for case-patients reporting severe disease compared with those reporting mild disease.

## Results

### Descriptive

NESSS clinical data were available for 3,241 STEC O157:H7 case-patients with genomic strain data in England during 2009–2019. Of those, 2,891 (89.2%) reported diarrheal symptoms, including 1,862 (57.5 %) who had experienced bloody diarrhea. HUS reportedly developed in 86 (2.6 %) case-patients. Thus, 1,889 (58.3%) case-patients in the dataset were categorized as having severe disease, although this proportion varied by Stx subtype (Table 1). Case-patients categorized as having mild disease accounted for 41.7 % of the dataset and included 110 asymptomatic persons. Over half (56.8 %) of case-patients in the dataset were female and 36.5 % were children <16 years of age. Severe disease was more frequently reported among female than male patients, although this difference was not significant (59.7 % vs. 56.4 %; p = 0.09), and among adults than among children (62.7% vs. 50.5 %; p<0.01).

**Table 1:** Disease severity of 3,241 clinical cases of Shiga toxin–producing *Escherichia coli* O157:H7 Stx subtype infection, by patient age, sex, and isolate Stx subtype, England, 2009–2019*

Variable	All cases, no. (%)	Mild illness, no. (%)†	Severe illness, no. (%)‡	HUS, no. (%)
All O157s	3,241	1,352 (41.7)	1,889 (58.3)	86 (2.7)
Age group				
Child	1,185 (36.5)	586 (49.5)	599 (50.5)	66 (5.6)
Adult	2,056 (63.5)	766 (37.3)	1,290 (62.7)	20 (1.0)
Sex				
F	1,841 (56.8)	742 (40.3)	1,099 (59.7)	54 (2.9)
M	1,400 (43.2)	610 (43.6)	790 (56.4)	32 (2.3)
Stx subtype				
stx2c stx1a	903 (28)	286 (31.7)	617 (68.3)	0
stx2c	675 (20.9)	535 (79.3)	140 (20.7)	2 (0.3)
stx2a	686 (21.3)	254 (37)	432 (63)	27 (3.9)
stx2a stx2c	829 (25.7)	240 (29)	589 (71)	50 (6.0)
stx1a	32 (1)	13 (40.6)	19 (59.4)	0
stx2a stx1a	51 (1.6)	9 (17.6)	42 (82.4)	0
stx2a stx2c stx1a	49 (1.5)	9 (18.4)	40 (81.6)	0
No Stx subtype§	16 (0.5)	6 (0.4)	10 (0.5)	0

*HUS, hemolytic uremic syndrome. †Asymptomatic or nonbloody diarrhea. ‡Bloody diarrhea, HUS, or death. §Isolates underwent whole-genome sequencing, but Stx subtype was not available.

Genomic typing data were available for isolates from 3,225 (99.5%) cases. Most (81.4%) isolates belonged to 5 specific clades within 5 sublineages: 1c (n = 789), IIa (n = 438), IIc (n = 932), I/II (n = 133), and IIb (n = 336). Infections with isolates in sublineage IIa were mostly attributed to a large outbreak associated with imported salad leaves in 2016 (*23*); the other 4 sublineages were associated with domestic acquisition of infection within the United Kingdom (Table 2) (*24*).

**Table 2:** Disease severity of 3,225 clinical cases of Shiga toxin–producing *Escherichia coli* O157:H7 Stx subtype infection, by isolate Stx subtype and sublineage, England, 2009–2019

Lineage, stx profile	All cases, no. (%)	Mild illness, no. (%)*	Severe illness, no. (%)†
Ic			
stx2c stx1a	2 (0.3)	0	2 (100)
stx2c	15 (1.9)	9 (60)	6 (40)
stx2a	309 (39.2)	97 (31.4)	212 (68.6)
stx2a stx2c	455 (57,7)	129 (28.4)	326 (71.6)
stx1a	1 (0.1)	0	1 (100)
stx2a stx1a	7 (0.9)	0	7 (100)
stx2a stx2c stx1a	0	0	0
I/II			
stx2c stx1a	0	0	0
stx2c	1 (0.8)	0	1 (100)
stx2a	82 (61.7)	9 (11)	73 (89)
stx2a stx2c	50 (37.6)	14 (28)	36 (72)
stx1a	0	0	0
stx2a stx1a	0	0	0
stx2a stx2c stx1a	0	0	0
IIb			
stx2c stx1a	5 (1.5)	2 (40)	3 (60)
stx2c	60 (17)	47 (78.3)	13 (21.7)
stx2a	257 (76.5)	134 (52.1)	123 (47.9)
stx2a stx2c	14 (4.2)	4 (28.6)	10 (71.4)
stx1a	0	0	0
stx2a stx1a	0	0	0
stx2a stx2c stx1a	0	0	0
Other			
stx2c stx1a	896 (7.8)	284 (41.9)	612 (58.1)
stx2c	599 (67.4)	479 (80.1)	120 (19.9)
stx2a	38 (3.4)	14 (21.1)	24 (78.9)
stx2a stx2c	310 (14.9)	93 (37.8)	217 (62.2)
stx1a	31 (1.6)	13 (66.7)	18 (33.3)
stx2a stx1a	44 (4.5)	9 (20)	35 (80)
stx2a stx2c stx1a	49 (0.4)	9 (0)	40 (0)

*Asymptomatic or nonbloody diarrhea. †Bloody diarrhea, hemolytic uremic syndrome, or death.

The dataset contained data for 86 case-patients with HUS, of which 32 were male and 54 were female. Most (66) HUS case-patients were children; infection progressed to HUS for 5.9% (66/1,119) of children, compared with 0.98% (20/2,039) of adults (Table 1).

### Severity by Subtype and Multiplicative Nature

The strains of STEC O157:H7 in this dataset had genes encoding Stx1a, Stx2a, or Stx2c, or combinations of those 3 subtypes (Table 1). Of those strains that harbored 1 *stx* subtype, those that had *stx1a* or *stx2a* were significantly more associated with severity than those that had *stx2c* (Tables 1–3). Comparisons of the *stx* subtype profiles exhibited by STEC O157:H7 indicated that strains with >1 *stx* subtype gene are associated with higher odds of severe disease than those with 1 *stx* subtype gene (Table 3). When *stx2c*, for which disease severity was lowest, was coupled with *stx1a*, the odds of severity increased (OR 7.89, 95% CI 6.23–9.97) to that comparable to strains possessing *stx2a* only (OR 7.04, 95% CI 5.51–9.00). The highest odds of severe disease were among case-patients infected with strains harboring *stx2a* and *stx1a* (OR 19.45, 95% CI 9.20–41.16).

**Table 3:** Univariate and multivariable regression analysis of disease severity for 3,225 patients with Shiga toxin–producing *Escherichia coli* O157:H7 infection, by isolate Stx subtype and patient age and sex, England, 2009–2019

Category	Univariate		Multivariable*	
	OR (95% CI)		OR (95% CI)	p value
Stx subtype				
stx2c stx1a	8.24 (6.53–10.40)		7.89 (6.23–9.97)	<0.001
stx2c	Referent 1.00		Referent	
stx2a	6.5 (5.10–8.28)		7.04 (5.51–9.00)	<0.001
stx2a stx2c	9.38 (7.38–11.91)		10.12 (7.94–12.90)	<0.001
stx1a	5.58 (2.69–11.58)		5.44 (2.61–11.36)	<0.001
stx2a stx1a	17.83 (8.48–37.51)		19.45 (9.20–41.16)	<0.001
stx2a stx2c stx1a	16.98 (8.05–35.83)		17.38 (8.20–36.86)	<0.001
Age group				
Adult	Referent 1.00		Referent	
Child	0.61 (0.3–0.70)		0.56 (0.48–0.66)	<0.001
Sex				
M	Referent 1.00		Referent	
F	1.14 (0.99–1.31)		1.09 (0.93–1.27)	0.286

*Adjusted for all other covariates in the model.

The most common *stx* profile in isolates from HUS case-patients was *stx2a/stx2c* (n = 50), followed by *stx2a* (n = 27) (Table 1). Only 2 HUS case-patients were infected with strains that did not have *stx2a* (both *stx2c* only). Five sublineages were represented among isolates from HUS case-patients: sublineage Ic (n = 54), sublineage IIa (n = 12), sublineage IIc (n = 6), lineage I/II (n = 12), and sublineage IIb (n = 2).

Subtype Stx2a is associated with 3 sublineages common in the United Kingdom: Ic, IIb, and I/II (Table 2). To explore the relationship between sublineage, clade, and severe disease, we conducted analysis by clade for Stx2a and Stx2a/2c. We found no significant difference in the odds of severity and clade for isolates encoding both Stx2a and Stx2c (Table 4). For isolates encoding Stx2a only, odds of severe symptoms were significantly higher for patients infected with isolates belonging to sublineage Ic and sublineage I/II than sublineage IIb (Table 4). Furthermore, isolates from only 2 HUS case-patients in the study belonged to sublineage IIb, despite the presence of Stx2a*.*

**Table 4:** Univariate and multivariable regression analysis of disease severity of Shiga toxin–producing *Escherichia coli* O157:H7 infection by sublineage, England, 2009–2019

Category	Univariate		Multivariable*	
	OR (95% CI)		OR (95% CI)	p value
*Stx2a* only strains, n = 648				
Sublineage				
IIb	Referent			
Ic	2.46 (1.75–3.46)		2.60 (1.83–3.677)	<0.001
I/II	9.09 (4.37–18.93)		8.91 (4.26–18.63)	<0.001
Age group				
Adult	Referent		Referent	
Child	0.65 (0.44–0.97)		0.62 (0.40–0.95)	0.03
Sex				
M	Referent		Referent	
F	1.10 (0.74–1.63)		1.16 (0.76–1.78)	0.50
*Stx2a/2c* strains, n = 505				
Sublineage				
Ic	Referent			
I/II	1.03 (0.54–1.98)		0.998 (0.521–1.91)	0.997
Age group				
Adult	Referent			
Child	0.77 (0.52–1.13)		0.79 (0.535–1.167)	0.237
Sex				
M	Referent			
F	1.3 (0.88–1.91)		1.267 (0.859–1.87)	0.232

*Adjusted for all other covariates in the model.

## Discussion

This large study of enhanced microbiological and epidemiologic data captured detailed clinical outcomes linked to molecular typing and phylogenetic analysis for adults and children infected with STEC O157:H7 in England. STEC O157:H7 is a rare but potentially very serious infection and particularly in children and elderly persons is likely to result in their interaction with healthcare services. Frontline laboratories have long had diagnostics in place and routinely screen all fecal specimens for STEC O157:H7. Therefore, NESSS captures data for a high proportion of STEC O157:H7 cases in England and is probably representative of STEC cases nationally.

Although our dataset is comprehensive, the potential for an inherent surveillance bias toward detecting more severe disease exists. Conversely, STEC HUS is underascertained in NESSS (PHE in-house data) because of challenges with the diagnosis of this condition. Moreover, for the most part, patient’s symptoms are self-reported; therefore, misclassification bias is possible, although because of the temporality of data collection, we consider bias to be low.

In our study, although HUS developed in more children <16 years of age, risk for severe disease seems to be lower than for those >16 years of age. It is possible that children are more likely to be taken for healthcare visits regardless of illness severity; therefore, our surveillance system is more likely to pick up milder cases of STEC infection in children than in adults.

Previous studies have documented the association between the presence of Stx2a and the development of HUS; thus, monitoring the presence and emergence of strains harboring this Stx subtype in the STEC population is needed (*12*–*17*,*19*). Most of these studies included STEC from a wide variety of different serotypes, exhibiting a variety of Stx subtypes and relatively small datasets. In contrast, we analyzed a large dataset, focusing on a single serotype characterized by limited number of Stx subtype combinations. Doing so enabled us to make direct comparisons between specific Stx profiles without the confounding influence of the wide variety of virulence factors expressed by different STEC serotypes.

Our analysis revealed that the acquisition of *stx1a* by STEC O157:H7 also increases the association with severity. This association is significant in strains of STEC O157:H7 *stx2c* that acquire *stx1a*; the odds of severe disease from strains harboring *stx1a/stx2c* are comparable to the odds of severe disease from strains that have *stx2a* when compared with *stx2c* only. This finding supports previous findings that serogroups other than STEC O157 harboring *stx1a* only have been isolated from patients reporting severe and prolonged gastrointestinal symptoms (e.g., STEC O117) (*25*). Cases of bloody diarrhea and HUS caused by STEC *stx1*-only strains do occur, albeit at a lower frequency than cases caused by STEC harboring *stx2* (*26*). However, the fact that *stx1a-*only isolates were not detected in our HUS cohort may support using presence of *stx2a* as a predictor of the highest likelihood of HUS development.

Analysis of the sublineages associated with HUS highlighted the rarity of sublineage IIb, despite increasing numbers of cases detected in the United Kingdom belonging to lineage IIb carrying *stx2a* (*14*). This finding correlates with the analysis showing that despite the presence of *stx2a*, isolates belonging to sublineage IIb are significantly less likely to be associated with severity than isolates belonging to sublineage 1c and I/II. These results indicate that the presence of *stx2a* is not the only driver behind HUS and that other factors are at play. These factors may include the *stx*-bacteriophage backbone, the *stx*-bacteriophage insertion site (*24*), copy number of the *stx2a* subtype gene, mutations in the *stx2a* subtype gene, or other gene mutations or deletions that may be involved in the expression of the toxin in vivo. A previous study (*27*) found that phylogenetic lineage seems to be predictive of HUS risk among those >10 years of age only and that lineage does not seem to explain HUS progression among children <10 years of age. They also observed that different lineages were observed at varying frequencies across age groups, suggestive of differences in exposure and acquisition of STEC.

This large study, which explored the association between STEC O157:H7 Stx subtype and disease severity in England over an 11-year period, provides further evidence that STEC O157:H7 exhibiting *stx* profiles that included *stx2a* only or in combination with other *stx* subtypes were more likely to be isolated from patients reporting bloody diarrhea, HUS, or both. However, we also observed that strains of STEC O157:H7 that had *stx1a* and *stx2a* only, or in combination with other *stx* subtypes, were significantly more associated with severe disease outcomes than those strains of STEC O157:H7 that had *stx2c* only. This finding confounds the clinical and public health risk assessment algorithms in many counties, including the United Kingdom, that are based on using detection of *stx2* as a predictor of severe gastrointestinal disease.
